# Polypropylene Nanocomposite Filled with Spinel Ferrite NiFe_2_O_4_ Nanoparticles and In-Situ Thermally-Reduced Graphene Oxide for Electromagnetic Interference Shielding Application

**DOI:** 10.3390/nano9040621

**Published:** 2019-04-16

**Authors:** Raghvendra Singh Yadav, Ivo Kuřitka, Jarmila Vilčáková, Michal Machovský, David Škoda, Pavel Urbánek, Milan Masař, Marek Gořalik, Michal Urbánek, Lukáš Kalina, Jaromir Havlica

**Affiliations:** 1 Centre of Polymer Systems, University Institute, Tomas Bata University in Zlín, Trida Tomase Bati 5678, 760 01 Zlín, Czech Republic; kuritka@utb.cz (I.K.); vilcakova@utb.cz (J.V.); machovsky@utb.cz (M.M.); dskoda@utb.cz (D.S.); urbanek@utb.cz (P.U.); masar@utb.cz (M.M.); murbanek@utb.cz (M.U.); 2 Faculty of Technology, Tomas Bata University in Zlín, Vavrečkova 275, 760 01 Zlín, Czech Republic; goralik@utb.cz; 3 Materials Research Centre, Brno University of Technology, Purkyňova 464/118, 61200 Brno, Czech Republic; kalina@fch.vut.cz (L.K.); havlica@fch.vut.cz (J.H.)

**Keywords:** nanoparticles, polymer-matrix composites, electrical properties, electromagnetic interference shielding

## Abstract

Herein, we presented electromagnetic interference shielding characteristics of NiFe_2_O_4_ nanoparticles—in-situ thermally-reduced graphene oxide (RGO)—polypropylene nanocomposites with the variation of reduced graphene oxide content. The structural, morphological, magnetic, and electromagnetic parameters and mechanical characteristics of fabricated nanocomposites were investigated and studied in detail. The controllable composition of NiFe_2_O_4_-RGO-Polypropylene nanocomposites exhibited electromagnetic interference (EMI) shielding effectiveness (SE) with a value of 29.4 dB at a thickness of 2 mm. The enhanced EMI shielding properties of nanocomposites with the increase of RGO content could be assigned to enhanced attenuation ability, high conductivity, dipole and interfacial polarization, eddy current loss, and natural resonance. The fabricated lightweight NiFe_2_O_4_-RGO-Polypropylene nanocomposites have potential as a high performance electromagnetic interference shielding nanocomposite.

## 1. Introduction

Nowadays, the advancement in electromagnetic interference (EMI) sensitive electronic devices, telecommunications, and radar systems has enforced the development of the outstanding electromagnetic interference shielding materials to be utilized to protect sensitive work places, in environmental protection appliances, and as anti-electromagnetic interference coatings [1]. In general, electromagnetic interference has several unwanted radiations that could create malfunction or noise in electronic appliances and also be detrimental to the human body [2]. To settle these issues, development of advanced shielding materials is in progress to achieve the requirements in the current industries. On the basis of the fundamental principles of electromagnetic interference shielding materials, the main mechanism involves reflection, multiple reflections, and absorption. In the absorption-dominant shielding material, the electrical conductivity and the electric dipole are important factors for electric materials, and the existence of magnetic dipoles is a critical factor for the magnetic materials [3]. 

Recently, carbon-based materials such as graphene have received tremendous scientific attention due to their excellent chemical and physical properties such as high specific surface area, outstanding electrical conductivity, thermal and mechanical characteristics, etc. [4,5,6]. The outstanding characteristics of graphene have led to the appearance of graphene as a potential nano-filler for the formation of advanced multifunctional nanocomposite materials [7]. However, the existence of a serious divergence in the values of the complex permittivity and permeability of carbon materials will prompt most of the electromagnetic wave to be reflected instead of absorbed [8]. According to the impedance matching approach, one of the active impressions to resolve this issue is to integrate with spinel ferrite magnetic nanoparticles. Therefore, anchoring spinel ferrite nanoparticles with graphene in a polymer system would be an effective approach to creating advanced EMI shielding nanocomposites. Recently, polypropylene has been received as a potential polymer matrix for magnetic and dielectric nanoparticle fillers to fabricate advanced nanocomposites for electromagnetic interference shielding applications. George G. et al. [9] reported nanocomposites of polypropylene in-situ reduced graphene oxide with outstanding electromagnetic interference shielding characteristics. Ameli A. et al. [10] reported that the lightweight polypropylene/stainless-steel fiber composite foam is excellent for electromagnetic interference shielding. In addition, Ameli A. et al. [11] reported the polypropylene/carbon fiber composite foams are outstanding for electromagnetic interference shielding applications. Hong M.-S. et al. [12] reported the carbon fibers in polypropylene matrix composites and their electromagnetic interference shielding characteristics. 

Further, a number of studies on EMI shielding properties of spinel ferrite with graphene have been reported by researchers [13,14,15]. However, there are few studies on EMI shielding properties of nanocomposites consisting of NiFe_2_O_4_ spinel ferrite nanoparticles with graphene. Zhang Y. et al. [16] demonstrated an easy approach of confined embedding of NiFe_2_O_4_ particles with graphene, and noticed highly tuned electromagnetic properties with tailored magnetic clusters. In another course of investigation, Liu P. et al. [17] reported that the morphology of NiFe_2_O_4_ particles has a significant impact on the microwave absorption characteristics. Ren F. et al. [18] studied the electromagnetic interference shielding characteristics of graphene nanosheets and nickel ferrite particles filled in cyanate ester. He J.-Z. et al. [19] prepared reduced graphene oxides in combination with nickel ferrite nanoparticles, and further investigated their electromagnetic properties in detail. In addition, Sabet M. et al. [20] reported graphene, NiFe_2_O_4_, and polypyrrole nanocomposites and studied their microwave absorption characteristics. Bateer B. et al. [21] synthesized NiFe_2_O_4_/graphene composite material and noticed excellent microwave absorption properties. Moreover, Wang Y. et al. [22] reported the NiFe_2_O_4_@SiO_2_@reduced graphene oxide composite as a potential electromagnetic interference shielding material. Further, Yan J. et al. [23] fabricated nanocomposite by conducting polymers such as polyaniline, polypyrrole, and poly (3,4-ethylenedioxythiophene) and with NiFe_2_O_4_ coated on reduced graphene oxide (RGO) sheets and noticed that prepared ternary composites exhibited outstanding microwave absorption characteristics. Furthermore, Liu P. et al. [24] fabricated composites consisting of nickel ferrite particles and graphene and noticed excellent microwave absorption characteristics. In our earlier study, our scientific group investigated the EMI shielding characteristics of lightweight NiFe_2_O_4_-reduced graphene oxide-elastomer nanocomposites [25]. The utilized magnetic nano-filler nickel ferrite was prepared by the honey assisted sol-gel combustion synthesis technique and reduced graphene oxides were attained through thermal reduction of graphene oxide at 500 °C in a tube furnace with atmosphere of argon. 

The EMI shielding effectiveness of nanocomposites depends on the preparation method and the preparation condition of the nano-fillers. In the present work, the in-situ thermally-reduced graphene oxide was employed as a dielectric filler in a polypropylene-based elastomer system containing NiFe_2_O_4_ spinel ferrite magnetic nanoparticles to prepare nanocomposite materials for electromagnetic interference shielding applications. The EMI shielding, permittivity, and permeability characteristics of the fabricated nanocomposite sheets were studied over the frequency 5.8 to 8.2 GHz. The existence and increase of the reduced graphene oxide contents in the elastomer system improved the EMI shielding characteristics of the prepared nanocomposites. The high aspect ratio and electrical conductivity of reduced graphene oxide sheets with excellent magnetic characteristics of nickel ferrite nanoparticles in polypropylene elastomer systems make them promising candidate for the preparation of lightweight and flexible sheets for EMI shielding applications. To the best of our awareness, this is the first time an easy preparation technique has been reported for the formation of polypropylene elastomer nanocomposites filled with spinel ferrite NiFe_2_O_4_ nanoparticles with in-situ reduced graphene oxide. In addition, the fabricated nanocomposites were characterized and studied in detail for their application as electromagnetic interference shielding materials. 

## 2. Materials and Methods 

### 2.1. Materials 

Nickel nitrate hexahydrate (Ni(NO_3_)_2_·6H_2_O) (98%) and iron nitrate nonahydrate (Fe(NO_3_)_3_·9H_2_O) (98%) were purchased from Alfa Aesar GmbH & Co., KG, Karlsruhe, Germany. The utilized starch (C_6_H_10_O_5_)_n_ was purchased from Lach-Ner, Neratovice, Czech Republic. Graphite flakes (Sigma-Aldrich, product no. 332461) and potassium permanganate (KMnO_4_) were purchased from Sigma Aldrich, Munich, Germany. The utilized sodium nitrate (NaNO_3_) was purchased from Lach-Ner, Neratovice, Czech Republic. Polypropylene elastomer as type Vistamaxx 6202 was purchased from Exxon Mobil, Machelen, Belgium. 

### 2.2. Preparation of NiFe_2_O_4_ Nanoparticles

The magnetic nano-filler NiFe_2_O_4_ spinel ferrite were prepared by the starch-mediated sol-gel combustion synthesis technique [26]. For the preparation of NiFe_2_O_4_ nanoparticles, nickel nitrate hexahydrate and iron nitrate nonahydrate were dissolved in 150 mL of deionized water to form a mixed solution. The molar ratio of the nickel nitrate to iron nitrate was 1:2. In the above mixed solution, the prepared 150 mL aqueous solution of 5 g starch was further added. The above formed mixed solution with the addition of the aqueous solution of starch was further placed on a hot plate with continuous magnetic stirring at a temperature of 110 °C. A viscous gel was formed after a few minutes with the evaporation of water from the mixed solution. In addition, the obtained gel was heated to the higher temperature 300 °C to get a combustion reaction and further formation of NiFe_2_O_4_ nanoparticles powder. 

### 2.3. Preparation of Graphene Oxide

The used graphene oxide for the formation of nanocomposites was prepared by modified Hummer’s method [27]. For preparation, 1.5 g graphite powder with 1.5 g sodium nitrate were mixed together. In the mixture, 75 mL concentrated sulphuric acid (H_2_SO_4_) was further added gently and further stirred for 15 min at room temperature. The mixed solution was retained in an ice bath. Further, 9 g potassium permanganate was added gently to the above mixture for about half hour. This obtained mixture was further stirred for 30 min at this temperature. Then, it was further stirred for 2 days at room temperature. 138 mL deionized water was added with stirring for 10 min, and as a result a thick paste was formed. Further, 420 mL warm deionized water and 30 mL hydrogen peroxide (H_2_O_2_) was added and further washed with an aqueous solution of sulphuric acid and hydrogen peroxide. The obtained yellow suspension was washed with deionized water until the pH was neutral.

### 2.4. Formation of Polypropylene Elastomer Nanocomposites Filled with Spinel Ferrite NiFe_2_O_4_ Nanoparticles and In-Situ Thermally-Reduced Graphene Oxide (RGO)

**Figure 1:**
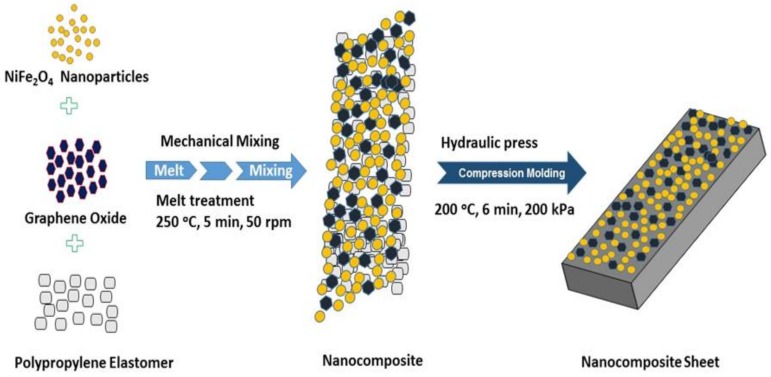
Schematic illustration of the preparation of polypropylene nanocomposites with spinel ferrite NiFe_2_O_4_ nanoparticles and in-situ thermally-reduced graphene oxide.

**Figure 2:**
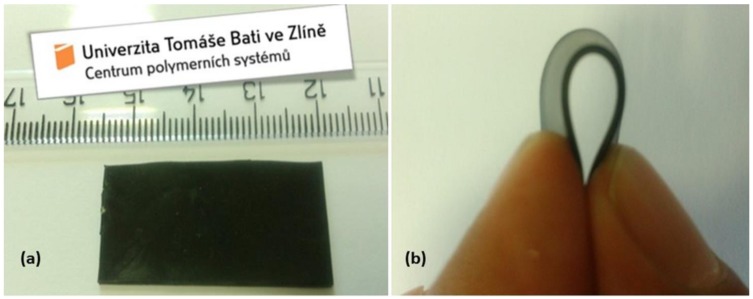
(**a**,**b**) Digital photograph of a prepared polypropylene nanocomposite with spinel ferrite NiFe_2_O_4_ nanoparticles and in-situ thermally-reduced graphene oxide.

NiFe_2_O_4_ nanoparticles were mixed with 1 wt%, 3 wt%, and 5 wt% graphene oxide by using mortars and pestles. A similar sample in the absence of graphene oxide was also prepared to note the influence of graphene oxide in nanocomposites. The propylene-based elastomer Vistamaxx 6202 was used as the polymer matrix for fabrication of nanocomposite samples. The prepared 50 wt% nano-filler was mixed with 50 wt% polypropylene-based elastomer as the polymer matrix for the preparation of nanocomposite samples. The nickel ferrite nanoparticles, graphene oxide, and elastomer were mixed together and compounded by a co-rotating conical twin screw extruder Micro-Compounder Xplore MC15. Further, the mixed material was melt treated at high a temperature of about 250 °C for 5 min at the speed of 50 rpm to achieve a higher level of dispersion. During this heat treatment process, the graphene oxide is in-situ thermally reduced [28,29,30]. In-situ reduction of graphene oxide at a moderate temperature in a polymer matrix is a different scenario in comparison with the reduction of graphene oxide in a furnace at a moderate temperature. The level of reduction of graphene oxide in different polymer matrices in air and at a low temperature (150–250 °C) depends on the chemistry of the polymer at the particles’ location and the processing history [31]. Figure 1 shows the schematic illustration of the formation of polypropylene nanocomposites with spinel ferrite NiFe_2_O_4_ nanoparticles and in-situ thermally reduced graphene oxide. After heat treatment, the four nanocomposite samples were formed, namely, (i) NiFe_2_O_4_-Polypropylene (ii) NiFe_2_O_4_-1wt%RGO-Polypropylene, (iii) NiFe_2_O_4_-3wt%RGO-Polypropylene, and (iv) NiFe_2_O_4_-5wt%RGO-Polypropylene. Moreover, the sheets of nanocomposite samples were formed by the hot press method. For this, the nanocomposites were molded in a hydraulic press (Fontijne LabEcon 300, Barendrecht, The Netherlands) at 200 °C for 6 min under pressure of 200 kPa with an average thickness of 2 mm. The prepared nanocomposite sheets (Figure 2) were used to measure the physical characteristics.

### 2.5. Characterization

The X-ray diffractometer Rigaku MiniFlex 600 (Rigaku Corporation, Tokyo, Japan) with a cobalt source (1.78897 Å for Co Kα) was utilized to characterize the crystal structure of the nanocomposites. The observation of the morphology of the nanoparticles was studied using a field emission scanning electron microscope (FEI NanoSEM450, Hillsboro, OR, USA). The field emission scanning electron microscope was utilized to investigate the cross section of composites prepared by freeze-fracturing in liquid nitrogen. Further, the morphology of nano-fillers and prepared nanocomposites was also investigated by the high-resolution transmission electron microscope JEOL JEM 2100 (JEOL, Peabody, MA, USA) with LaB_6_ cathode operated at an acceleration voltage of 200 kV. The cross sections of polypropylene elastomer (Vistamax) materials with added NiFe_2_O_4_ nanoparticles and reduced graphene oxide for TEM were done by ultramicrotomy using ultramicrotome Leica EM UC7 with cryochamber at −120 °C. Powder samples for TEM imaging were dispersed in water (18.2 Mohm.cm) in an ultrasonic bath for 10 min. The X-ray photoelectron spectra of nanoparticles was obtained by using a Kratos Analytical Ltd., (Manchester, UK). Raman Spectra were measured at 532 nm by using a Raman microscope Nicolet DXR (Thermo Fisher Scientific, Waltham, MA, USA). The complex permittivity, permeability, and electromagnetic parameters of prepared nanocomposites were studied with the vector network analyzer Agilent N5230A (Agilent Technologies, Santa Clara, CA, USA). The frequency range of the vector network analyzer was 5.8 to 8.2 GHz. The FTIR measurements were performed with the help of Nicolet 6700 (Thermo Scientific, Waltham, MA, USA). The magnetic characteristics were investigated with a vibrating sample magnetometer (VSM, Model 7407, Lake Shore, Westerville, OH, USA). Mechanical properties were studied by using the Testometric universal-testing machine (Testometric Co., Ltd., Rochdale, UK). For mechanical measurement, the dumb-bell-shaped specimens were prepared from the fabricated nanocomposite sheets with a punching press. The observed data of the mechanical performances are the average results of at least five tests.

## 3. Results

### 3.1. X-ray Diffraction Study

**Figure 3:**
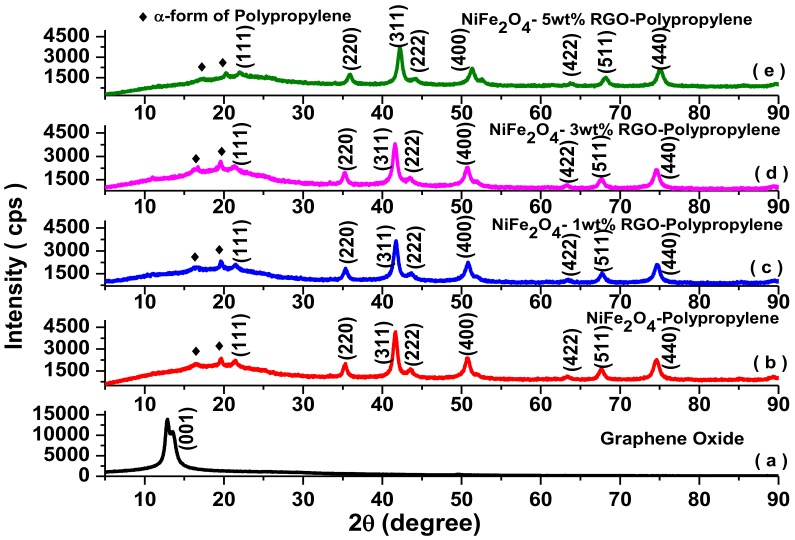
X-ray powder diffraction patterns of (**a**) graphene oxide, (**b**) NiFe_2_O_4_-Polypropylene (**c**) NiFe_2_O_4_-1wt%RGO-Polypropylene, (**d**) NiFe_2_O_4_-3wt%RGO-Polypropylene, and (**e**) NiFe_2_O_4_-5wt%RGO-Polypropylene.

Figure 3 depicts the X-ray diffraction pattern of graphene oxide, NiFe_2_O_4_-Polypropylene, NiFe_2_O_4_-1wt%RGO-Polypropylene, NiFe_2_O_4_-3wt%RGO-Polypropylene, and NiFe_2_O_4_-5wt%RGO-Polypropylene nanocomposite samples. As shown in Figure 3a, the X-ray diffraction peak of graphene oxide appears at 2θ = 12.8°, which reveals an increase in the interlayer distance of graphene sheets due to the presence of oxygen-functionalized chemical groups. The polypropylene-based elastomer nanocomposites (Figure 3b–e) show characteristic diffraction peaks at 2θ = 21.4°, 35.3°, 41.7°, 43.6°, 50.8°, 63.4°, 67.6°, and 74.8° corresponding to (111), (220), (311), (222), (400), (422), (511), and (440) cubic spinel ferrite phases of NiFe_2_O_4_ nanoparticles, respectively [32]. In addition, polypropylene-based elastomer nanocomposites show characteristic peaks at 2θ = 16.6° and 19.6° corresponding to the crystalline planes of the α-form of polypropylene [33,34]. Further, in the obtained X-ray diffraction pattern of NiFe_2_O_4_-RGO-Polypropylene nanocomposites, the diffraction peaks related to the restacking of in-situ reduced graphene oxide could not be detected, revealing that the polypropylene elastomer nanocomposites efficiently prevented the restacking of in-situ reduced graphene oxide [35]. 

### 3.2. Field Emission Scanning Electron Microscopy (FE-SEM) and Transmission Eelectron Microscopy (TEM) Study

**Figure 4:**
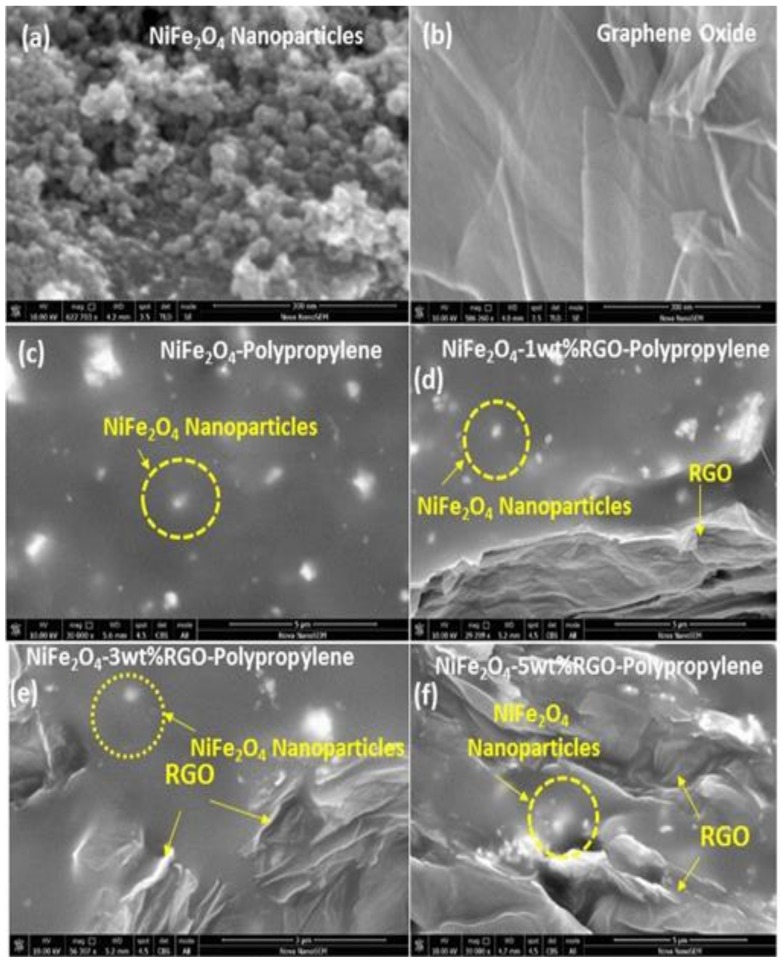
Field emission scanning electron microscopy (FE-SEM) images of (**a**) spinel ferrite NiFe_2_O_4_ nanoparticles and (**b**) prepared graphene oxide, and cross sections of (**c**) NiFe_2_O_4_-Polypropylene, (**d**) NiFe_2_O_4_-1wt% RGO-Polypropylene, (**e**) NiFe_2_O_4_-3wt% RGO-Polypropylene, and (**f**) NiFe_2_O_4_-5wt%RGO-Polypropylene nanocomposite.

**Figure 5:**
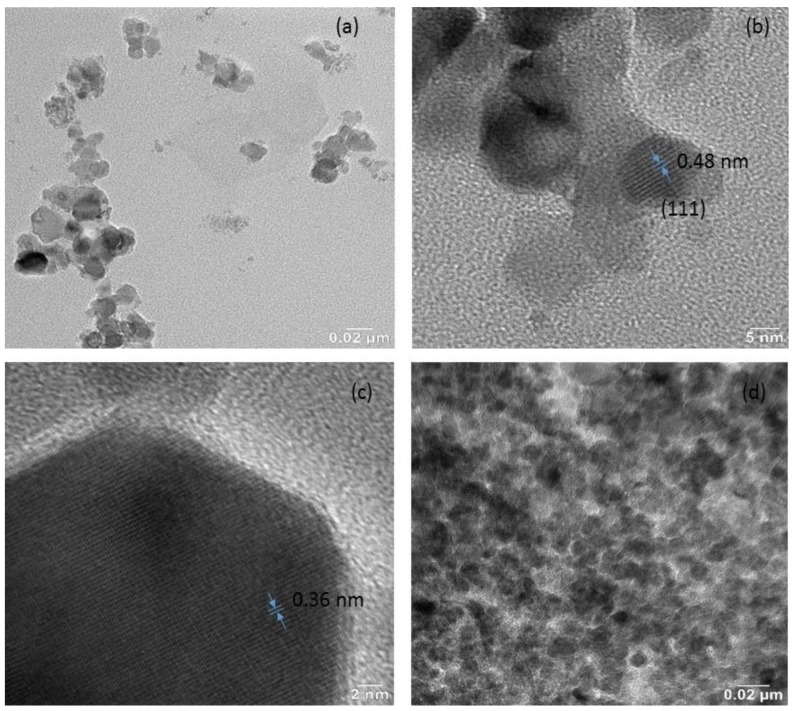
(**a**) TEM study of NiFe_2_O_4_ nanoparticles, (**b**) HRTEM study of NiFe_2_O_4_ nanoparticles, (**c**) HRTEM study of graphene oxide, and (**d**) TEM study of NiFe_2_O_4_-5wt%RGO-Polypropylene elastomer nanocomposites.

Figure 4a shows the field emission scanning electron microscopy (FE-SEM) image of NiFe_2_O_4_ nanoparticles. The synthesized nanoparticles have spherical morphology with particle size 20–60 nm. Figure 4b shows the FE-SEM image of prepared graphene oxide. The figure depicts a wrinkled paper-like surface of the graphene oxide sheets. Further, Figure 4c depicts the FE-SEM micrograph of the cross section of a polypropylene elastomer nanocomposite containing NiFe_2_O_4_ nanoparticles. It shows that in the polypropylene elastomer the nanoparticles of NiFe_2_O_4_ are uniformly distributed. Furthermore, Figure 4d–f shows the FE-SEM image of the cross section of polypropylene nanocomposites with in-situ reduced graphene oxide and NiFe_2_O_4_ nanoparticles. It can be noticed from Figure 4d–f that the in-situ reduced graphene oxide and NiFe_2_O_4_ spinel ferrite nanoparticles exist in the polypropylene system, indicating the formation of hybrid nanocomposites, which is also evident from the Fourier transform infrared spectroscopy (FTIR) and Raman investigation. Herein, the SEM images (Figure 4c–f) of the cross section of the nanocomposites were measured by using a back-scattered electron detector at accelerating voltage 10 kV. In addition, the SEM image of the cross section of the nanocomposite using a secondary electron detector (accelerating voltage 5 kV) can be seen in Figure S1 (Supplementary Materials). Further, TEM and HRTEM studies were carried out to obtain a closer morphology and structure of the NiFe_2_O_4_ nanoparticles, graphene oxide, and polypropylene nanocomposites. The TEM image (Figure 5a) of NiFe_2_O_4_ nanoparticles shows particles of 20–60 nm. Figure 5b depicts an HRTEM image of NiFe_2_O_4_ nanoparticles, in which some individual nanoparticles display clear crystal lattice of spacing 0.48 nm corresponding to (111) crystal plane. Figure 5c depicts the HRTEM image of graphene oxide, which indicates lattice fringes of a graphene oxide sheet. The separation of neighboring fringes was 0.36 nm. In addition, the low-resolution TEM image (Figure 5d) of NiFe_2_O_4_-5wt%RGO-Polypropylene elastomer nanocomposite exhibits that NiFe_2_O_4_ nanoparticles with reduced graphene oxide are highly dispersed in the polypropylene elastomer matrix, which supports the FE-SEM observations.

### 3.3. Fourier Transform Infrared Spectroscopy (FTIR) Study

**Figure 6:**
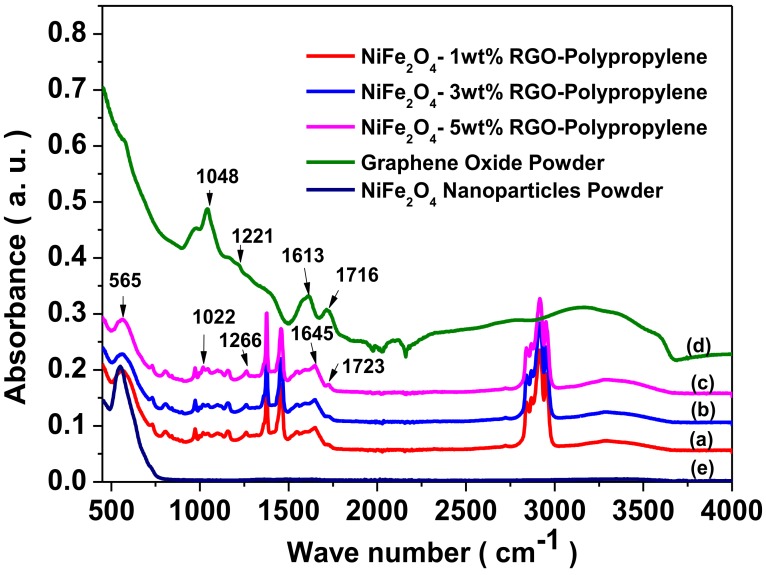
Fourier transform infrared spectroscopy (FTIR) spectra of (a) NiFe_2_O_4_-1wt%RGO-Polypropylene, (b) NiFe_2_O_4_-3wt%RGO-Polypropylene, (c) NiFe_2_O_4_-5wt%RGO-Polypropylene, (d) prepared graphene oxide powder, and (e) NiFe_2_O_4_ nanoparticles.

Fourier transform infrared spectroscopy (FTIR) is utilized to investigate chemical bonding or the physical entanglement that occurs among the NiFe_2_O_4_ nanoparticles, in-situ reduced graphene oxide, and polypropylene-based elastomer. Figure 6 depicts FTIR spectra of NiFe_2_O_4_-1wt%RGO-Polypropylene, NiFe_2_O_4_-3wt%RGO-Polypropylene, NiFe_2_O_4_-5wt%RGO-Polypropylene, graphene oxide powder, and spinel ferrite NiFe_2_O_4_ nanoparticles. As shown in Figure 6a–c, the major absorption bands in polypropylene-based elastomer nanocomposites are at 2750–3000 cm^−1^ and 1300–1500 cm^−1^ and are associated with the vibration of chemical species such as C-H and -CH_3_ of polypropylene, respectively. This is in good agreement with the previous reports by other researchers on polypropylene composites [36,37]. The intensities of the FTIR peaks related to oxygen functionalities, such as the C=O stretching vibration band at 1716 cm^−1^ in graphene oxide (Figure 6d), decrease to a significant extent after the in-situ reduction of graphene oxide with a vibration band at 1723 cm^−1^ (Figure 6a–c). The C-O (epoxy) stretching vibration band at 1221 cm^−1^ of graphene oxide was changed to 1266 cm^−1^ due to the in-situ reduction of graphene oxide. In addition, the C-O (alkoxy) stretching vibration band at 1048 cm^−1^ of graphene oxide was changed to 1022 cm^−1^. The band at 1613 cm^−1^ is related to the skeletal vibration of unoxidized graphitic domains [38]. In polypropylene nanocomposites, all the chemical bands associated with the oxygen containing functional groups in graphene oxide nearly diminished, and revealed the removal of these attached chemical groups during the formation of nanocomposites at processing temperature 250 °C. This observation confirms the in-situ reduction of graphene oxide during the formation of polypropylene nanocomposite [39]. The vibrational band at 565 cm^−1^ is related to the intrinsic stretching vibration of metal at tetrahedral sites in NiFe_2_O_4_ spinel ferrite nanoparticles. This observation confirms the existence of NiFe_2_O_4_ spinel ferrite nanoparticles in polypropylene-based elastomer nanocomposites.

### 3.4. Raman Spectra Study

**Figure 7:**
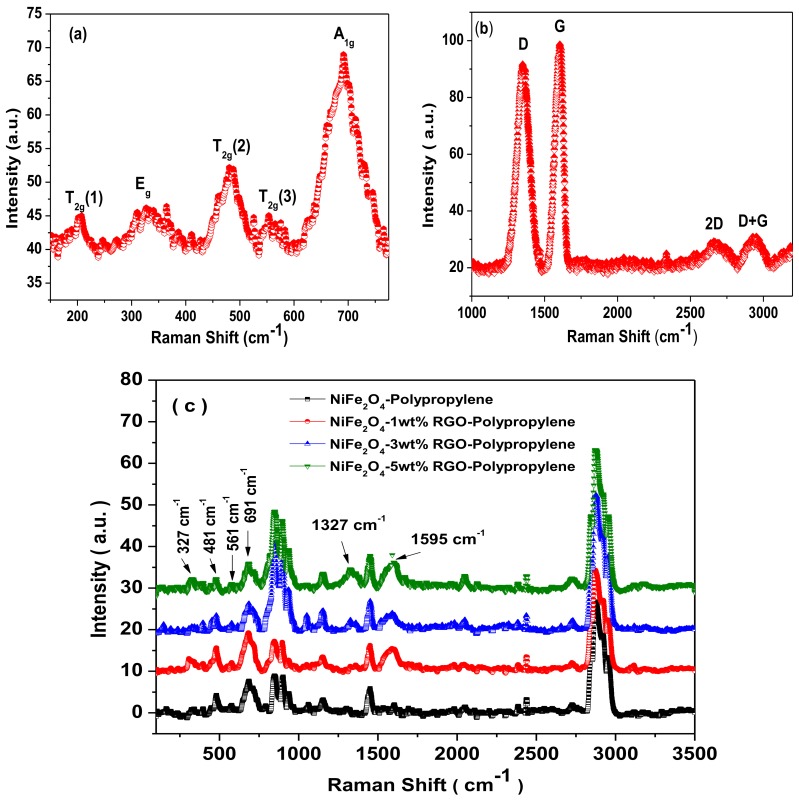
Raman spectra of (**a**) NiFe_2_O_4_ nanoparticles, (**b**) Graphene Oxide, and (**c**) Polypropylene nanocomposites with NiFe_2_O_4_ nanoparticles and in-situ reduced graphene oxide.

Raman spectroscopy is a non-destructive characterization tool utilized for confirmation of the formation of the crystal structure of spinel ferrite. Figure 7a represents the Raman spectrum of NiFe_2_O_4_ spinel ferrite nanoparticles. The factor group analysis provides five Raman active bands (A_1g_ + E_g_ + 3T_2g_) for cubic spinel ferrite [40]. The Raman spectrum of synthesized NiFe_2_O_4_ nanoparticles exhibited five Raman bands at 693 cm^−1^, 324 cm^−1^, 205 cm^−1^, 481 cm^−1^, and 552 cm^−1^ as shown in Figure 7a. In Figure 7a, the Raman band at 693 cm^−1^ is A_1g_ mode associated with symmetric stretch of a tetrahedral iron-oxygen ion in the spinel ferrite [41]. The Raman band at 324 cm^−1^ is E_g_ mode associated with the symmetric bending of an oxygen-metal ion at the octahedral site in the spinel ferrite. Further, the Raman band at 205 cm^−1^ is T_2g_(1) mode due to asymmetric bending of the oxygen ion with respect to the metal ion at the octahedral site in the spinel ferrite. Furthermore, the Raman band at 481 cm^−1^ is T_2g_(2) mode related to the asymmetric stretching of the oxygen metal ion at the octahedral site. Further, the Raman band at 552 cm^−1^ is T_2g_(3) and corresponds to the translation movement of the oxygen with respect to the iron ion [42]. Figure 7b depicts the Raman spectra of graphene oxide, which was utilized in preparation of nanocomposite samples. The Raman spectra of graphene oxide exhibited the D-band at 1350 cm^−1^, the G-band at 1601 cm^−1^, the 2D-band at 2665 cm^−1^, and the D+G band at 2935 cm^−1^. The observed D-band corresponds to the random arrangement of graphite or lead by lattice defects of carbon atoms. The observed G-band and 2D-band are related to the stretching vibration of carbon atoms in the sp^2^-hybridized plane and the secondary Raman scattering of the regional boundary phonon, respectively [43]. Furthermore, the observed D+G combination band is related to the existence of disorder in the graphene oxide [44].

Figure 7c depicts Raman spectra of prepared nanocomposites. The Raman modes corresponding to the NiFe_2_O_4_ spinel ferrite crystal structure can be noticed at 327 cm^−1^, 481 cm^−1^, 561 cm^−1^, and 691 cm^−1^. The D-band and G-band corresponding to the reduced graphene oxide can be noticed at 1327 cm^−1^ and 1595 cm^−1^, respectively. It can be noticed that the position of G-band in the nanocomposite is shifted by 6 cm^−1^ to 1595 cm^−1^ from 1601 cm^−1^. The shift in G-band is associated with the reduction of graphene oxide in the nanocomposite. A smaller Raman shift of 6 cm^−1^ signifies a lesser degree of reduction of graphene oxide with a C:O ratio less than 10 [31]. Furthermore, the other observed Raman bands between 800 cm^−1^ and 3500 cm^−1^ in Figure 7c are associated with polypropylene [45,46]. 

### 3.5. X-ray Photoelectron Spectroscopy (XPS) Study

**Figure 8:**
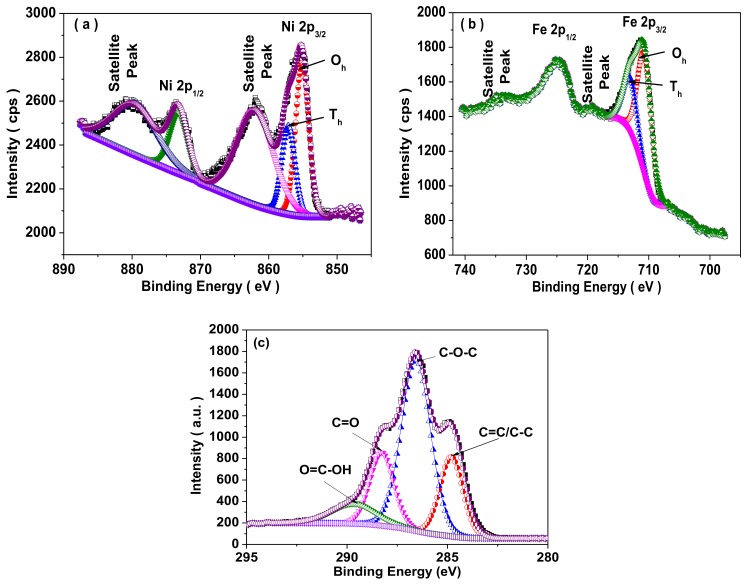
X-ray photoelectron spectroscopy (XPS) spectrum with deconvoluted peaks of (**a**) Ni for prepared NiFe_2_O_4_, (**b**) Fe for NiFe_2_O_4_, and (**c**) C 1s for graphene oxide, utilized in preparation of nanocomposite samples.

Figure 8a,b shows the X-ray photoelectron spectroscopy (XPS) of NiFe_2_O_4_ spinel ferrite nanoparticles. In Figure 8a, the Ni 2p XPS spectra exhibited two binding peaks assigned as Ni 2p_3/2_ at 855.2 eV and Ni 2p_1/2_ at 873.5 eV with satellite peaks at 862.2 eV and 880.2 eV. It reflects the existence of Ni^2+^ in NiFe_2_O_4_ nanoparticles. Moreover, the asymmetric nature of Ni 2p_3/2_ peak reflects the existence of Ni^2+^ ions at octahedral and tetrahedral sites in prepared NiFe_2_O_4_ spinel ferrite nanoparticles. The Ni^2+^ ions at the octahedral site (O_h_) and the tetrahedral site (T_h_) were 63% and 37%, respectively [47]. In Figure 8b, the presence of two binding energy peaks Fe 2p_3/2_ at 711.1 eV and Fe 2p_1/2_ at 724.9 eV with satellite peaks at 719.3 eV and 734.4 eV, confirms the existence of Fe^3+^ ions in NiFe_2_O_4_ nanoparticles. The satellite peaks are associated with the transition of an electron from a 3d orbital to the vacant 4s orbital at the time of the ejection of the core 2p photoelectron [48]. The evaluated value indicates that the Fe^3+^ were 68% and 32% at the octahedral (O_h_) and tetrahedral sites (T_h_), respectively [49]. Figure 8c depicts the high resolution XPS spectrum of graphene oxide, which was utilized in preparation of nanocomposite samples. The sp^2^ peak (C=C/C-C) of the C1s XPS spectrum is noticed at 284.8 eV. Further, the binding energy peaks at 286.5 eV, 288.2 eV, and 289.6 eV related to the epoxide group (C-O-C), carbonyl (C=O) and carboxyl (O=C-OH) group, respectively [50].

### 3.6. Magnetic Property

**Figure 9:**
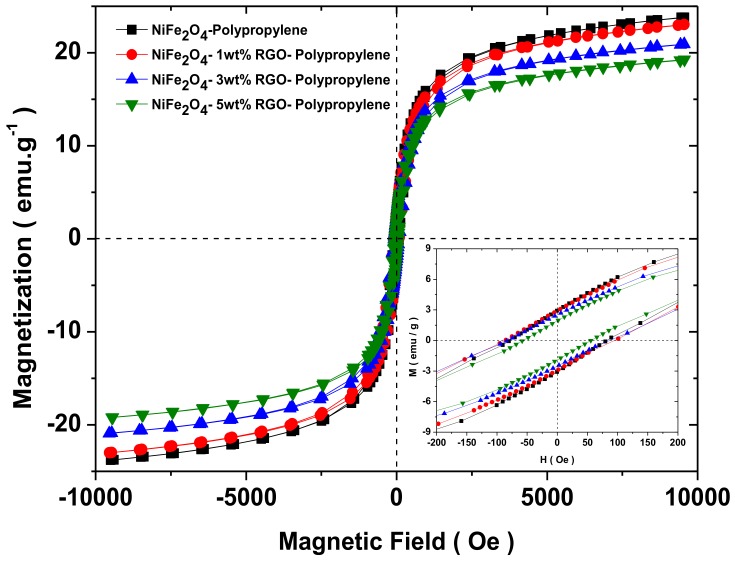
Magnetic hysteresis curves of polypropylene nanocomposites filled with spinel ferrite NiFe_2_O_4_ nanoparticles and in-situ reduced graphene oxide (RGO). Inset is enlarged view.

The magnetic characteristics of prepared nanocomposites were investigated by vibrating sample magnetometer. Figure 9 depicts the magnetic hysteresis curves of prepared nanocomposites. From Figure 9, it can be seen that the prepared nanocomposites exhibited ferromagnetic behaviour. Further, Table 1 reflects the saturation magnetization (M_s_), coercivity (H_c_), and remanent magnetization (M_r_) values evaluated from Figure 9. The saturation magnetization of prepared nanocomposite samples decreases with the increase of the RGO filler due to the existence of a nonmagnetic RGO filler. 

### 3.7. Electromagnetic Interference Shielding Effectiveness and Electromagnetic Parameters 

**Figure 10:**
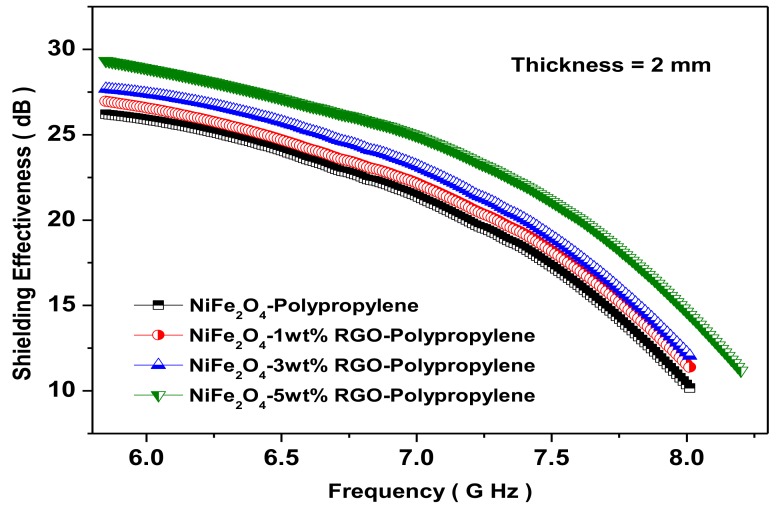
Electromagnetic interference shielding effectiveness (dB) for NiFe_2_O_4_-Polypropylene, NiFe_2_O_4_-1wt% RGO-Polypropylene, NiFe_2_O_4_-3wt%RGO-Polypropylene, and NiFe_2_O_4_-5wt%RGO-Polypropylene elastomer nanocomposite samples at thickness 2 mm.

The new and ingeniously prepared polypropylene-based elastomer nanocomposites filled with spinel ferrite NiFe_2_O_4_ nanoparticles of good magnetic properties and reduced graphene oxide of moderate electrical conductivity could be potentially utilized as shielding materials. Figure 10 depicts the total shielding effectiveness (*SE_T_*) of prepared nanocomposites with a frequency in the range of 5.8–8.2 GHz. The total shielding effectiveness *SE_T_* for shielding materials is [51]:

$$SE_{T}\left(\mathrm{dB}\right)=SE_{R}\left(\mathrm{dB}\right)+SE_{A}\left(\mathrm{dB}\right)+SE_{M}\left(\mathrm{dB}\right)$$

where *SE_R_*, *SE_A_*, and *SE_M_* are the shielding effectiveness related to reflection, absorption, and multiple reflections, respectively. Here, the term related to multiple reflections *SE_M_* can be flouted when *SE_T_* > 10 dB as [52]:

$$SE_{T}\left(\mathrm{dB}\right)=SE_{R}\left(\mathrm{dB}\right)+SE_{A}\left(\mathrm{dB}\right)$$

The *SE_T_* is evaluated by using scattering parameters measured from a vector network analyzer. The measured scattering parameters have a correlation with the reflectance *R* and the transmittance *T* as [53]:

$$R={\left|S_{11}\right|}^{2}={\left|S_{22}\right|}^{2}$$

$$T={\left|S_{12}\right|}^{2}={\left|S_{21}\right|}^{2}$$

which provide absorbance as *A* = (1 − *R* − *T*). Further, the *SE_R_* and *SE_A_* have the following relations in terms of the reflectance R and the transmittance *T* [54]:

$$SE_{R}=-10\log_{10}\left(1-R\right)$$

$$SE_{A}=-10\log_{10}\left[T/\left(1-R\right)\right]$$

The shielding effectiveness depends on the magnetic and dielectric properties related to the following relations [55]:

$${SE}_{R}\left(\mathrm{dB}\right)\approx 10\log\left(\sigma_{ac}/\left(16{\omega \varepsilon }_{o}\mu_{r}\right)\right)$$

and

$$SE_{A}\left(dB\right)=20t\sqrt{\frac{\mu_{r}\omega \sigma_{ac}}{2}}=8.68\left(\frac{t}{\delta }\right)$$

where *ε_o_*, *μ_r_*, *ω*, and *σ_ac_* are the free space permittivity, the relative magnetic permeability, the angular frequency, and the electrical ac conductivity, respectively. The above relations indicate that the *SE_R_* decreases, and the *SE_A_* increases with the increase of the applied frequency. The term *SE_R_* is associated with the value of (*σ_ac_/μ_r_*), which suggests that the *SE_R_* is larger for the higher conductivity and smaller magnetic permeability of the material. Further, the term *SE_A_* is related to the value of *μ_r_σ_ac_*, which suggest that the value of *SE_A_* is higher for material with higher electrical conductivity and higher magnetic permeability.

**Figure 11:**
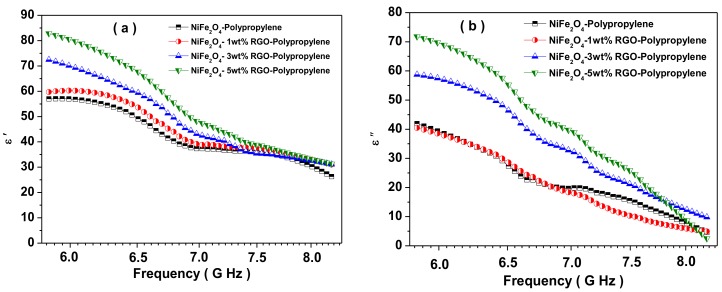

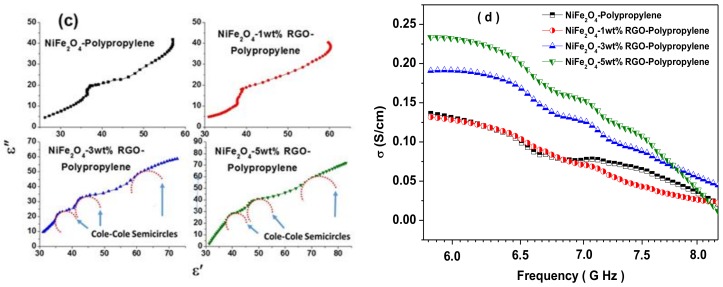
(**a**) Real part of the permittivity of the prepared nanocomposite, (**b**) imaginary part of the permittivity of the prepared nanocomposite, (**c**) Cole–Cole plots for the prepared nanocomposite, (**d**) AC conductivity of the prepared nanocomposite.

The total shielding effectiveness (*SE_T_*) of NiFe_2_O_4_-Polypropylene, NiFe_2_O_4_-1wt%RGO-Polypropylene, NiFe_2_O_4_-3wt%RGO-Polypropylene, and NiFe_2_O_4_-5wt%RGO-Polypropylene nanocomposite samples are 26.1, 26.9, 27.7, and 29.4 dB, respectively, at 5.8 GHz. This indicates that the value of SE_T_ of the nanocomposites increases with an increase in the content of reduced graphene oxide (RGO). Pawar, S.P. et al. [56] studied EMI SE characteristics of PC (polycarbonate)/SAN[poly(styrene-co-acrylonitrile)] nanocomposites with few-layered graphene nanosheets and nickel nanoparticles (G-Ni). A total shielding effectiveness (SET) of 29.4 dB at 18 GHz was noticed for this nanocomposite. Further, Saini, P. et al. [57] reported EMI SE of poly(aniline)-coated fabrics containing dielectric and magnetic nanoparticles. This research group observed that pure poly(aniline)-coated fabric exhibited a total shielding effectiveness (*SE_T_*) of 15.3 dB and that it was enhanced to 16.8 dB and further to 19.4 dB with a filling of BaTiO_3_ and Fe_3_O_4_ nanoparticles, respectively. In addition, Chen, Y. et al. [58] investigated the EMI SE characteristics of polyaniline composites incorporated with graphene decorated by metallic nanoparticles. This research group work demonstrated that both the electrical conductivity and the EMI SE were increased with filler loading, and the EMI SE value of 29.33 dB was achieved. For aerospace application, weight of shielding material is an important design parameter, therefore, the specific EMI shielding effectiveness (EMI SE divided by density) is evaluated. The specific EMI shielding effectiveness (SSE) was 38.59 dB·cm^3^g^−1^, 40.00 dB·cm^3^g^−1^, 43.02 dB·cm^3^g^−1^, and 46.77 dB·cm^3^g^−1^ for NiFe_2_O_4_-Polypropylene, NiFe_2_O_4_-1wt%RGO-Polypropylene, NiFe_2_O_4_-3wt% RGO-Polypropylene, and NiFe_2_O_4_-5wt% RGO-Polypropylene elastomer nanocomposites, respectively. These observed values of SSE are much higher than those of typical metals (10 dB·cm^3^g^−1^ for solid copper), carbon nanotube-polystyrene foam composite (33.1 dB·cm^3^g^−1^), etc. [59]. Further, the SSE is not a sufficient design parameter because a higher value of SSE can be received at a high thickness, and, consequently, there is a direct increase in the weight of the shielding material. Therefore, absolute EMI shielding effectiveness (SSE divided by material thickness) is a more realistic design parameter [60]. The absolute shielding effectiveness (SSE/t) was 192.9 dB·cm^2^g^−1^, 200.0 dB·cm^2^g^−1^, 215.1 dB·cm^2^g^−1^, and 233.86 dB·cm^2^g^−1^ for NiFe_2_O_4_-Polypropylene, NiFe_2_O_4_-1wt%RGO-Polypropylene, NiFe_2_O_4_-3wt% RGO-Polypropylene, and NiFe_2_O_4_-5wt% RGO-Polypropylene elastomer nanocomposites, respectively. Furthermore, we studied in detail the permittivity (*ε*) and permeability (*μ*) of the prepared nanocomposite sheets. The real part of the permittivity and permeability is directly associated with the content of polarization generated in the prepared nanocomposite, which signifies the storage ability of the electric energy and magnetic energy, respectively. In addition, the imaginary part of the permittivity and permeability signifies the dissipation of electric energy and magnetic energy, respectively. Figure 11a depicts the real part of the permittivity (*ε*′) of the prepared nanocomposites with the different weight ratio of RGO. The values of *ε*′ of nanocomposites are in the range of 56.7–25.8, 59.8–30.0, 72.1–30.7, and 83.1–31.8 for NiFe_2_O_4_-Polypropylene, NiFe_2_O_4_-1wt%RGO-Polypropylene, NiFe_2_O_4_-3wt%RGO-Polypropylene, and NiFe_2_O_4_-5wt%RGO-Polypropylene nanocomposites, respectively. In addition, the values of *ε*′ of nanocomposites increases with the increase of the nano-filler content RGO. The value of ε′ is the result of the polarization of the material, which is related to the dipole and the interface polarizations under the electromagnetic field. Moreover, due to the existence of the residual bonds and defects, the electrons are not uniformly distributed, which generate the orientation polarization, and consequently enhancement in the value of *ε*′ [61].

Figure 11b depicts the imaginary part (*ε*″) of the permittivity with the different weight ratio of RGO in nanocomposites. The values of *ε*″ are in the range of 42.0–4.4, 40.6–5.3, 58.6–9.8, and 72.1–2.4 for NiFe_2_O_4_-Polypropylene, NiFe_2_O_4_-1wt%RGO-Polypropylene, NiFe_2_O_4_-3wt%RGO-Polypropylene, and NiFe_2_O_4_-5wt%RGO-Polypropylene nanocomposites, respectively. The value of *ε*″ of the nanocomposite increases with the increase of RGO filler content. It is associated with the formation of the conducting network by filling of the RGO content. Furthermore, the relative permittivity has the following relation [62]:

$$\varepsilon_{r}=\varepsilon_{\infty }+\frac{\varepsilon_{s}-\varepsilon_{\infty }}{1+j2\pi f\tau }=\varepsilon^{\prime }-j\varepsilon^{\prime \prime }$$

where, *ε_s_*, *ε_∞_*, *f*, and *τ* are the static permittivity, relative dielectric permittivity at the high frequency limit, frequency, and the relaxation time, respectively. Therefore, the real part of the permittivity (*ε*′) and the imaginary part of the permittivity (*ε*″) can be expressed as [63]:

$$\varepsilon^{\prime }=\varepsilon_{\infty }+\frac{\varepsilon_{s}-\varepsilon_{\infty }}{1+{\left(2\pi f\right)}^{2}\tau^{2}}$$

$$\varepsilon^{\prime \prime }=\frac{2\pi f\tau \left(\varepsilon_{s}-\varepsilon_{\infty }\right)}{1+{\left(2\pi f\right)}^{2}\tau^{2}}$$

From the above equations, *ε*′ and *ε*″ have a relationship, which can be expressed as [64]:

$${\left(\varepsilon^{\prime }-\frac{\varepsilon_{s}-\varepsilon_{\infty }}{2}\right)}^{2}+{\left(\varepsilon^{\prime \prime }\right)}^{2}={\left(\frac{\varepsilon_{s}-\varepsilon_{\infty }}{2}\right)}^{2}$$

Hence, the graph between *ε*′ and *ε*″ would be a single semicircle, and it is known as the Cole–Cole semicircle. Further, Figure 11c depicts the *ε*′ versus *ε*″ Cole–Cole plots for prepared nanocomposites. It is noticeable that the *ε*′ versus *ε*″ plots exhibited multiple semicircles for RGO loaded 1, 3, and 5 wt% nanocomposites. This indicates that the nanocomposites exhibit multiple relaxation processes, which are associated with the contribution of dipole and interfacial polarization to enhance the permittivity of nanocomposites with the increase of RGO content [65]. In addition, the electrical AC conductivity values of prepared nanocomposite samples were evaluated based on the following relation [66]:

$$\sigma_{AC}=2\pi f\varepsilon_{o}\varepsilon^{\prime \prime }$$

where *f*, *ε_o_*, and *ε*″ are the frequency, the permittivity of the free space, and the imaginary part of the permittivity, respectively. Figure 11d depicts the variation of the electrical conductivity of prepared nanocomposites. It can be seen in Figure 11d that the electrical conductivity was enhanced with the further addition of RGO nano-filler content in nanocomposite samples. The enhanced value of electrical conductivity with the RGO filler content signifies the existence of migration, tunnelling, and hopping phenomenon in the prepared nanocomposites.

**Figure 12:**
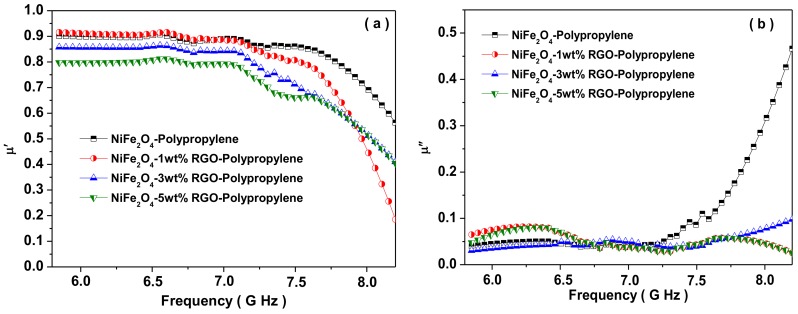

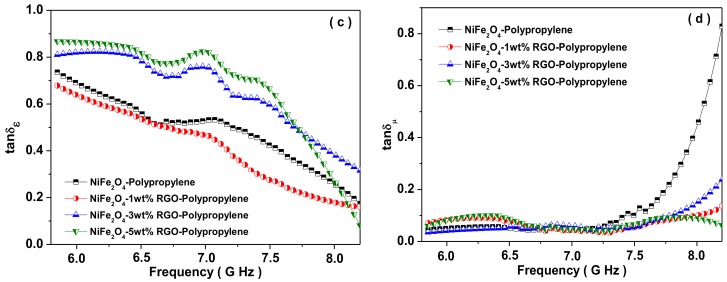
(**a**) Real part of the permeability, (**b**) imaginary part of the permeability, (**c**) dielectric loss tanδ_ε_, and (**d**) magnetic loss tan *δ_μ_* for NiFe_2_O_4_-Polypropylene, NiFe_2_O_4_-1wt%RGO-Polypropylene, NiFe_2_O_4_-3wt% RGO-Polypropylene, and NiFe_2_O_4_-5wt% RGO-Polypropylene elastomer nanocomposites.

Figure 12a depicts the real part of the permeability (*μ*′) of the prepared nanocomposites with different weight ratios of RGO nano-filler content. The values of μ′ are in the range of 0.89–0.56, 0.91–0.19, 0.86–0.42, and 0.80–0.41 for NiFe_2_O_4_-Polypropylene, NiFe_2_O_4_-1wt%RGO-Polypropylene, NiFe_2_O_4_-3wt%RGO-Polypropylene, and NiFe_2_O_4_-5wt%RGO-Polypropylene nanocomposites, respectively. Further, Figure 12b represents the imaginary part of the permeability (*μ*″) of the prepared nanocomposites with increased content of RGO nano-filler. The values of *μ*″ are in the range of 0.04–0.46, 0.06–0.03, 0.02–0.09, and 0.05–0.03 for NiFe_2_O_4_-Polypropylene, NiFe_2_O_4_-1wt%RGO-Polypropylene, NiFe_2_O_4_-3wt%RGO-Polypropylene, and NiFe_2_O_4_-5wt%RGO-Polypropylene nanocomposites, respectively. Furthermore, the dielectric loss (tan *δ_ε_* = *ε*″/*ε*′) and magnetic loss (tan *δ_μ_* = *μ*″/*μ*′) signify the extent of the quantity of electromagnetic wave energy loss compared to the quantity of the electromagnetic wave energy storage, respectively. Herein, the dielectric loss (tan δ_ε_ = *ε*″/*ε*′) and magnetic loss (tan *δ_μ_* = *μ*″/*μ*′) of the prepared nanocomposites were projected by using the obtained permittivity and permeability parameters of the nanocomposites. Figure 12c depicts the tan *δ_ε_* values, which are less than 1 due to higher conductivity and the polarization of the prepared nanocomposites. It was observed that the dielectric loss of the nanocomposite with the 5 wt% RGO nano-filler is higher than those of other nanocomposites, which signifies that the 5 wt% RGO filled nanocomposite had an excellent electromagnetic wave energy loss capability, suggesting that RGO nano-filler played an important role in further increasing the dielectric loss. In addition, these observations could be associated with the large content of interfaces and thereby the interfacial polarization generated among surfaces of NiFe_2_O_4_, RGO, and elastomer. The dielectric loss mainly originates from dipole, electronic, and interfacial polarization and relaxation. The filler NiFe_2_O_4_ nanoparticle acted as very small dipoles, which got polarized in the existence of the electromagnetic wave and consequently electromagnetic wave absorption. Further, the NiFe_2_O_4_ nanoparticles with RGO sheets acted as a polarized centre in the elastomer matrix, which resulted in enhanced electromagnetic wave absorption. Furthermore, the existence of interfaces among NiFe_2_O_4_ nanoparticles, RGO sheets, and elastomer layers were answerable for interfacial polarization and further contributed to the dielectric losses. 

Figure 12d represents the tan *δ_μ_* values of nanocomposites, which are less than one for NiFe_2_O_4_-Polypropylene, NiFe_2_O_4_-1wt%RGO-Polypropylene, NiFe_2_O_4_-3wt%RGO-Polypropylene, and NiFe_2_O_4_-5wt%RGO-Polypropylene nanocomposites. The origin of magnetic loss is eddy current loss, hysteresis loss, domain wall resonance loss, natural resonance, etc. [67]. The hysteresis loss of NiFe_2_O_4_ nanoparticles in nanocomposite elastomer samples can be ignored in the investigated frequency range (weak field). The domain wall resonance loss of NiFe_2_O_4_ nanoparticles happens at a lower MHz frequency range. Hence, the eddy current loss and the natural resonance could be answerable for the electromagnetic wave attenuation. The eddy current loss has the following relation [68]:

$$\mu^{\prime \prime }\approx 2\;\pi \mu_{o}{\left(\mu^{\prime }\right)}^{2}\sigma d^{2}f/3$$

$$C_{o}=\mu^{\prime \prime }{\left(\mu^{\prime }\right)}^{-2}f^{-1}=2\;\pi \mu_{o}\sigma d^{2}/3$$

where *σ* and *μ*_o_ are the electrical conductivity and the permeability in a vacuum. If the origin of the magnetic loss is the eddy current loss, the values of *C_o_*(*C_o_* = *μ*″·(*μ*′)^−2^*f*^−1^) are constant with variation of the frequency.

**Figure 13:**
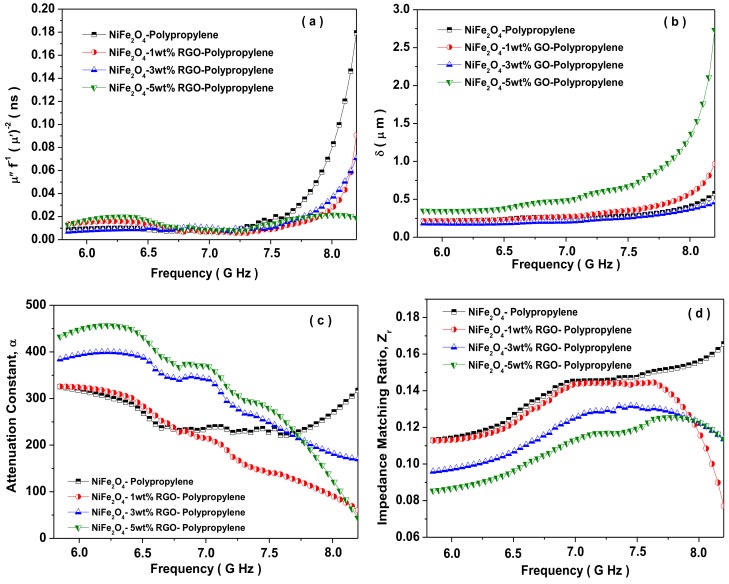
(**a**) Eddy current loss, (**b**) skin depth, (**c**) attenuation constant, (**d**) impedance matching for NiFe_2_O_4_-Polypropylene, NiFe_2_O_4_-1wt%RGO-Polypropylene, NiFe_2_O_4_-3wt%RGO-Polypropylene, and NiFe_2_O_4_-5wt%RGO-Polypropylene elastomer nanocomposites.

Figure 13a depicts the change of C_o_ value with frequency for prepared nanocomposites. It is noticeable from Figure 13a that the value of C_o_ remains approximately constant over 6.6–7.2 GHz frequency range. Hence, the magnetic loss of the nanocomposites over the frequency range 6.6–7.2 GHz is associated with the eddy current loss, whereas the magnetic loss over the frequency range 5.8–6.6 GHz and 7.2–8.2 GHz is caused by natural resonance. In addition, the natural resonance loss has the following relation [69]:

$$2\pi f_{r}=rH_{a}$$

$$H_{a}=4\left|K_{1}\right|/3\mu_{o}M_{s}$$

where *r*, *H_a_*, |*K*_1_|, and *M_s_* are the gyromagnetic ratio (2.8 GHz·kOe^−1^), the anisotropic energy, the anisotropic coefficient, and the saturation magnetization, respectively. As observed in Figure 9, the *M_s_* value of the RGO-loaded nanocomposite was decreased with the increase of RGO content, indicating an increase in anisotropy energy, which was beneficial to enhancing the attenuation of the EM waves at high frequency. Meanwhile, skin depth is an important factor for shielding material. The skin depth (*δ*) can be calculated by the following relation [70]:

$$\delta ={\left(\pi \sigma f\mu ’\right)}^{-1/2}$$

where *σ*, *μ*′, and *f* are the frequency-dependent conductivity, the real part of the permeability, and the frequency (Hz), respectively. The above relation suggests that the value of the conductivity (*σ*) is inversely proportional to the square of skin depth (*δ*^2^), therefore, a huge decrease in the skin depth of the shielding materials occurs under a slight increase of conductivity value. Consequently, there is an enhancement in the absorption of the incident electromagnetic wave. In addition, the presence of gaps between graphene sheets plays a vital role in generating the internal multiple reflection of the electromagnetic waves and thereby more absorption of the electromagnetic wave. Figure 13b depicts the change of skin depth (*δ*) of prepared nanocomposites with the frequency. It is noticeable in Figure 13b that the skin depth (*δ*) is constant in the lower frequency range, which signifies the existence of surface conduction at a lower frequency range. Further, to understand the EMI SE of the nanocomposites, the attenuation constant and impedance matching were investigated. The attenuation constant α is an important factor for an outstanding shielding nanocomposite. The attenuation constant signifies the capability for the dielectric loss and magnetic loss [71]. The attenuation constant α has the following relation [72]:

$$\alpha =\frac{\sqrt{2}\pi f}{c}\sqrt{\left(\mu^{\prime \prime }\varepsilon^{\prime \prime }-\mu^{\prime }\varepsilon^{\prime }\right)+\sqrt{{\left(\mu^{\prime \prime }\varepsilon^{\prime \prime }-\mu^{\prime }\varepsilon^{\prime }\right)}^{2}+{\left(\mu^{\prime }\varepsilon^{\prime \prime }-\mu^{\prime \prime }\varepsilon^{\prime }\right)}^{2}}}$$

where *f* and *c* are the frequency of electromagnetic wave propagation and the velocity of light, respectively. Figure 13c depicts the change of the attenuation constant *α* of prepared nanocomposites. The increased value of the electrical conductivity with the RGO nano-filler in nanocomposites facilitates the large attenuation constant. The 5 wt% RGO loaded nanocomposite sample has the highest attenuation constant *α*, leading to the most outstanding shielding nanocomposite material. The larger attenuation constant originates from more dielectric loss, which is helpful for EM wave absorbance performance [73]. Furthermore, the impedance matching behaviour also directly influences the EMI shielding effectiveness properties. In addition, the impedance matching ratio has the following relation [74]:

$$Z_{r}=Z/Z_{o}=\sqrt{\mu_{r}/\varepsilon_{r}}$$

where *Z*, *Z_o_*, *μ_r_*, and *ε_r_* are the impedance value of the absorbing material, the impedance of the free space, the complex permeability, and the complex permittivity, respectively. The impedance matching ratio signifies the capability of the electromagnetic waves to flow into the nanocomposite material. Figure 13d depicts the impedance matching ratio of the prepared nanocomposites with the frequency. It is noticeable in Figure 13d that the impedance matching ratio decreases with an increase of RGO filler content in the prepared nanocomposite samples. Further, the nanocomposite sample without and with 1 and 3 wt% RGO filler exhibited a high impedance matching ratio and the lowest attenuation constant due to low complex permittivity. However, the prepared nanocomposite with the further addition of a nano-filler of 5 wt% RGO possessed a low impedance matching ratio and a high attenuation constant. Under the impedance mismatch condition, a high content of incident electromagnetic waves get reflection back to the surface of the material, and therefore, a high value of reflection. On the other hand, if the attenuation ability is not high, the propagating wave cannot be fully attenuated. Because of the offset and balance between the attenuation constant and impedance matching ratio, the nanocomposite samples with 5 wt% RGO filler have enhanced electromagnetic interference shielding effectiveness property. Similar reports are also available which support the theory that the higher attenuation constant plays a more vital role in EMI shielding than impedance matching [75,76].

**Figure 14:**
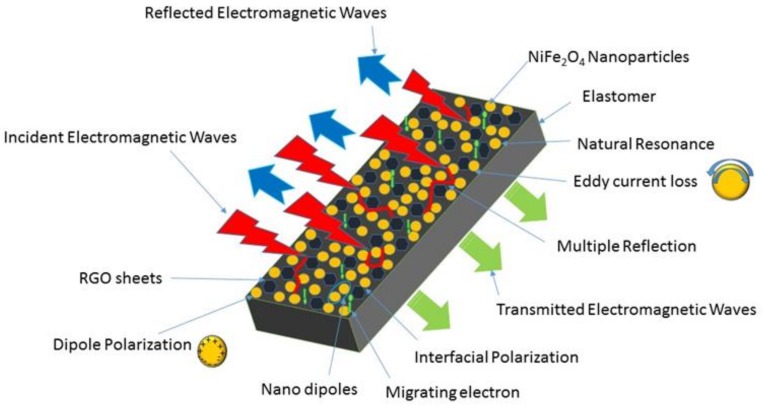
Schematic illustration of the possible mechanism of the electromagnetic wave shielding for polypropylene elastomer nanocomposites filled with spinel ferrite NiFe_2_O_4_ nanoparticles with in-situ reduced graphene oxide (RGO).

In accordance with the above results and analysis, the possible electromagnetic interference shielding mechanism can be explained with the help of Figure 14, which depicts the schematic illustration of the probable mechanism of the electromagnetic wave shielding for NiFe_2_O_4_-RGO-Elastomer nanocomposites. The incident electromagnetic waves propagate onto the surface of the nanocomposite thin film sample, one part of the incident electromagnetic energy enters into the nanocomposite sample and exhibits the occurrence of an interaction with the available electrons and charged particles, and some energy is dissipated as heat. Further, an electric part of the incident electromagnetic waves interacts with the NiFe_2_O_4_ nanoparticles and RGO, which behave as a polarization center. In addition, NiFe_2_O_4_ with RGO favor the enhancement of interfacial polarization. The dipole and interfacial relaxation process enhances the attenuation of the electromagnetic waves. The existence of RGO plays a vital role in enhancing the conductivity of the nanocomposite sample and forms the conducting network in the sample. The filling of RGO with NiFe_2_O_4_ nanoparticles in the elastomer matrix introduces interfacial polarization, which is probably related to the synergistic behavior in permittivity and conductivity with an increase in the RGO filler content [77]. The existence of abundant surface functional groups and lattice defects on ferrite nanoparticles and reduced graphene oxide and also the multiple interfaces on ferrite-graphene polypropylene generates multiple reflections and also the scattering of incident electromagnetic waves, and consequently enhances the electromagnetic absorption ability [78,79,80]. The magnetic part of the electromagnetic waves interacts with the magnetic NiFe_2_O_4_ nanoparticles and generates eddy current loss, magnetic tangent loss, and natural resonance. The enhanced attenuation ability and conductivity make the NiFe_2_O_4_-RGO-Elastomer nanocomposite a lightweight and an outstanding electromagnetic interference shielding material. 

### 3.8. Mechanical Properties 

**Figure 15:**
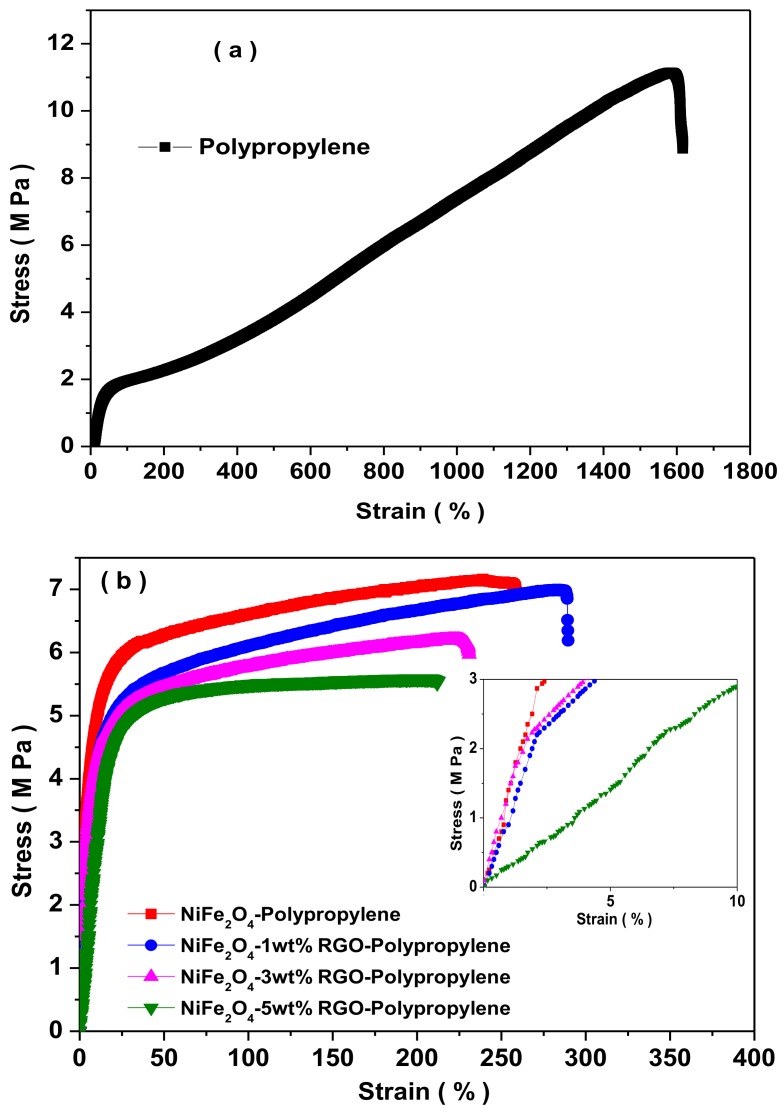
Strain–stress behavior of (**a**) polypropylene and (**b**) prepared polypropylene nanocomposites.

Figure 15 depicts the stress–strain behavior of pure polypropylene and its prepared polypropylene nanocomposites with NiFe_2_O_4_ spinel ferrite nanoparticles and reduced graphene oxide. It reveals the variation of stress–strain behavior with nano-fillers in polypropylene nanocomposites. The tensile strength was 7.31 MPa, 7.43 MPa, 6.56 MPa, and 5.03 MPa, for NiFe_2_O_4_-Polypropylene, NiFe_2_O_4_-1wt%RGO-Polypropylene, NiFe_2_O_4_-3wt%RGO-Polypropylene, and NiFe_2_O_4_-5wt%RGO-Polypropylene nanocomposites, respectively; while the tensile strength of utilized polypropylene is 11.9 MPa. Table 2 summarizes the measured tensile strength, Young’s modulus, and the elongation at the break of the prepared nanocomposite samples. The measured Young’s modulus was 30.01 MPa, 26.28 MPa, 25.29 MPa, and 22.34 MPa for NiFe_2_O_4_-Polypropylene, NiFe_2_O_4_-1wt%RGO-Polypropylene, NiFe_2_O_4_-3wt%RGO-Polypropylene, and NiFe_2_O_4_-5wt%RGO-Polypropylene nanocomposites, respectively. However, the Young’s modulus of utilized polypropylene is 2.7 MPa. The prepared nanocomposites showed a significant improvement in the Young’s modulus compared with pure polypropylene elastomer. The elongation at break (%) was 247.70%, 391.08%, 345.78%, and 250.25%, for NiFe_2_O_4_-Polypropylene, NiFe_2_O_4_-1wt%RGO-Polypropylene, NiFe_2_O_4_-3wt%RGO-Polypropylene, and NiFe_2_O_4_-5wt%RGO-Polypropylene nanocomposites, respectively. Furthermore, the elongation at break for the utilized polypropylene was 1695 %.

## 4. Conclusions

In this work, we fabricated polypropylene nanocomposites filled with spinel ferrite NiFe_2_O_4_ nanoparticles and in-situ reduced graphene oxide (RGO). The utilized nano-filler NiFe_2_O_4_ spinel ferrite nanoparticles were synthesized by the starch-mediated sol-gel combustion synthesis technique. The structural, morphological, magnetic, and electromagnetic parameters of prepared nanocomposites were evaluated by XRD, FE-SEM, TEM, Raman, FTIR, XPS, a vibrating sample magnetometer, and a vector network analyzer. The proper electromagnetic parameters with the different content of RGO result in the increased value of the attenuation ability. We believe that the fabricated NiFe_2_O_4_-RGO-Polypropylene elastomer nanocomposites with a well-controlled weight fraction of RGO can be applied as lightweight electromagnetic interference shielding materials.

## Figures and Tables

**Table 1:** Saturation magnetization (M_s_), remanent magnetization (M_r_), and coercivity (H_c_) of prepared nanocomposite samples.

Sample	M_s_ (emu/g)	M_r_ (emu/g)	H_c_ (Oe)
NiFe_2_O_4_-Polypropylene	24.0	3.0	83.1
NiFe_2_O_4_-1wt%RGO-Polypropylene	23.2	2.8	94.0
NiFe_2_O_4_-3wt%RGO-Polypropylene	20.9	2.4	88.2
NiFe_2_O_4_-5wt%RGO-Polypropylene	19.3	1.9	57.5

**Table 2:** Mechanical property parameters: Tensile strength, Young’s modulus, elongation at the break of prepared composite materials.

Sample	Tensile Strength (MPa)	Young’s Modulus (MPa)	Elongation at Break (%)
NiFe_2_O_4_-Polypropylene	7.31 ± 0.12	30.01 ± 1.43	247.70 ± 30.91
NiFe_2_O_4_-1wt%RGO-Polypropylene	7.43 ± 0.38	26.28 ± 1.14	391.08 ± 34.46
NiFe_2_O_4_-3wt%RGO-Polypropylene	6.56 ± 0.32	25.29 ± 1.74	345.78 ± 42.43
NiFe_2_O_4_-5wt%RGO-Polypropylene	5.03 ± 0.23	22.34 ± 1.34	250.25 ± 40.65

## Supplementary Materials

The following are available online at https://www.mdpi.com/2079-4991/9/4/621/s1, Figure S1: SEM images of cross section of (a) NiFe_2_O_4_-Polypropylene (b) NiFe_2_O_4_-1wt%RGO-Polypropylene, (c) NiFe_2_O_4_-3wt%RGO-Polypropylene, and (d) NiFe_2_O_4_-5wt%RGO-Polypropylene.
