# Supplementary material for: Polypropylene Nanocomposite Filled with Spinel Ferrite NiFe2O4 Nanoparticles and In-Situ Thermally-Reduced Graphene Oxide for Electromagnetic Interference Shielding Application
Source: Nanomaterials (Basel). 2019 Apr 16;9(4):621. doi: 10.3390/nano9040621 (PMC6523113; doi:10.3390/nano9040621)
Supplement: Supplementary file 1 [file nanomaterials-09-00621-s001.pdf]

Supporting Information

Polypropylene Nanocomposite Filled with Spinel Ferrite $NiFe_2O_4$ Nanoparticles and In-Situ Thermally-Reduced Graphene Oxide for Electromagnetic Interference Shielding Application

Raghvendra Singh Yadav [1,*], Ivo Kuřitka [1], Jarmila Vilčáková [1], Michal Machovský [1], David Škoda [1], Pavel Urbánek [1], Milan Masař [1], Marek Gořalik [2], Michal Urbánek [1], Lukáš Kalina [3], and Jaromir Havlica [3]

1 Centre of Polymer Systems, University Institute, Tomas Bata University in Zlín, Trida Tomase Bati 5678, 760 01 Zlín, Czech Republic; kuritka@utb.cz (I.K.); vilcakova@utb.cz (J.V.); machovsky@utb.cz (Michal Machovsky); dskoda@utb.cz (D.S.); urbanek@utb.cz (P.U.); masar@utb.cz (Milan Masař); murbanek@utb.cz (M.U.)

2 Faculty of Technology, Tomas Bata University in Zlín, Vavrečkova 275, 760 01 Zlín, Czech Republic; goralik@utb.cz

3 Materials Research Centre, Brno University of Technology, Purkyňova 464/118, 61200 Brno, Czech Republic; kalina@fch.vut.cz (L.K.); havlica@fch.vut.cz (J.H.)

* Correspondence: yadav@utb.cz; Tel: +420-576031725

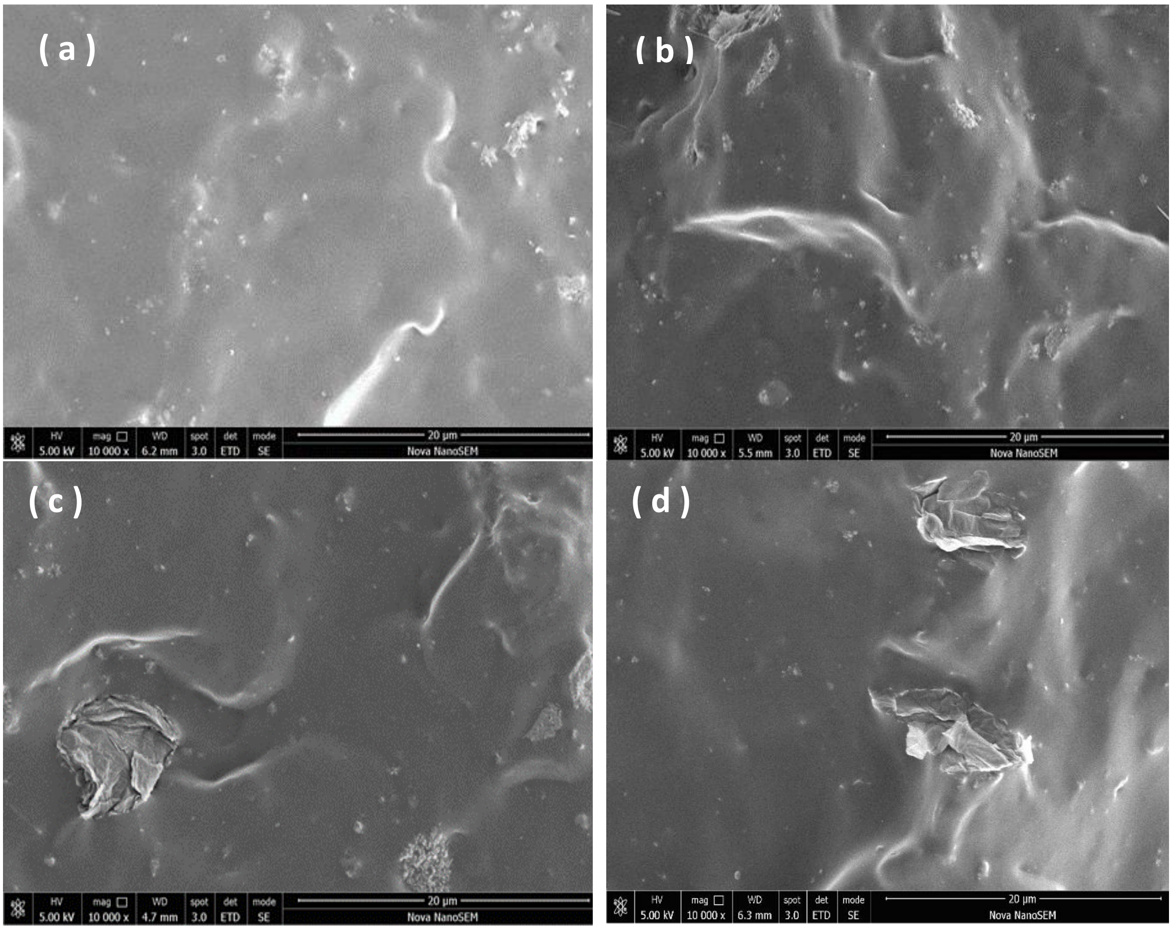

Figure S1. SEM images of cross-section of (**a**) $NiFe_2O_4$-Polypropylene (**b**) $NiFe_2O_4$-1wt%RGO-Polypropylene, (**c**) $NiFe_2O_4$-3wt%RGO-Polypropylene, (**d**) $NiFe_2O_4$-5wt%RGO-Polypropylene.
